# Undetected Ocular Mass as a Critical Alert for Identifying Uveal Metastasis

**DOI:** 10.1155/2024/5522370

**Published:** 2024-10-16

**Authors:** Tea Štrbac, Biljana Kuzmanović Elabjer, Antun Koprivanac, Mladen Bušić

**Affiliations:** ^1^University Eye Department, University Hospital “Sveti Duh”, Zagreb, Croatia; ^2^Reference Center of the Ministry of Health of the Republic of Croatia for Pediatric Ophthalmology and Strabismus, Zagreb, Croatia; ^3^Reference Center of the Ministry of Health of the Republic of Croatia for Inherited Retinal Dystrophies, Zagreb, Croatia; ^4^Reference Center of the Ministry of Health of the Republic of Croatia for Standardized Echography in Ophthalmology, Zagreb, Croatia; ^5^Faculty of Medicine, Josip Juraj Strossmayer University of Osijek, Osijek, Croatia; ^6^Faculty of Dental Medicine and Health Osijek, Josip Juraj Strossmayer University of Osijek, Osijek, Croatia; ^7^Clinic of Internal Medicine, University Hospital “Sveti Duh”, Zagreb, Croatia

**Keywords:** intraocular metastases, metastatic lung adenocarcinoma, undetected ocular mass

## Abstract

**Purpose:** We report our experience of diagnosing and managing metastatic adenocarcinoma of the lungs with primary manifestation to the iris and the ciliary body with the purpose to raise clinical suspicion for systemic malignancy in the presence of undetected ocular mass along with other ocular manifestations.

**Observations:** An 82-year-old male presented with a deterioration of vision and intense pain in the right eye since the day before. With his right eye, he suddenly discerned hand movements only. Intraocular pressure in the right eye was increased. Slit–lamp biomicroscopy revealed a ciliary injection and hemorrhagic mass overlying the temporal half of the iris. The L-9 ultrasound biomicroscopy (UBM) scan documented the tumor completely infiltrating the iris and the ciliary body. We suspected a metastatic eye lesion. CT chest imaging showed a solid expansive formation of the right lung. At the Oncology Department, a fine needle aspiration biopsy was performed under the control of MSCT, which confirmed lung adenocarcinoma.

**Conclusions and Importance:** Although very rarely, pathological changes in the uvea may indicate a metastatic occurrence that needs to be considered in the differential diagnosis. It is important to undergo a wide ophthalmological examination, which in this case included a standardized A-scan echography and UBM, which confirmed the suspicion of a tumor lesion, followed by cooperation with other medical professionals in order to discover a primary diagnosis.

## 1. Introduction

Uveal metastases stand as the predominant intraocular malignancy, frequently serving as the initial indicator of tumor spread [1]. In the majority of cases, intraocular metastasis affects the choroid due to the abundant vascular supply where metastases can be easily spread hematogenously, while anterior uveal metastases (to the iris and ciliary body) are only present in 10% of all uveal metastases [2]. Blurred vision or loss of vision, pain, floaters, visual field defects, and photophobia may indicate metastatic lesions of the iris or ciliary body [3]. Uveal metastatic lesions occur in approximately 2%–9% of patients with systemic solid malignant diseases (Stage IV disease). In adults, the most common metastatic tumors of the uvea are present in patients with extended breast cancer in the first place and lung cancer in the second place in a total percentage between 71% and 92% [4].

## 2. Case Presentation

**Figure 1:**
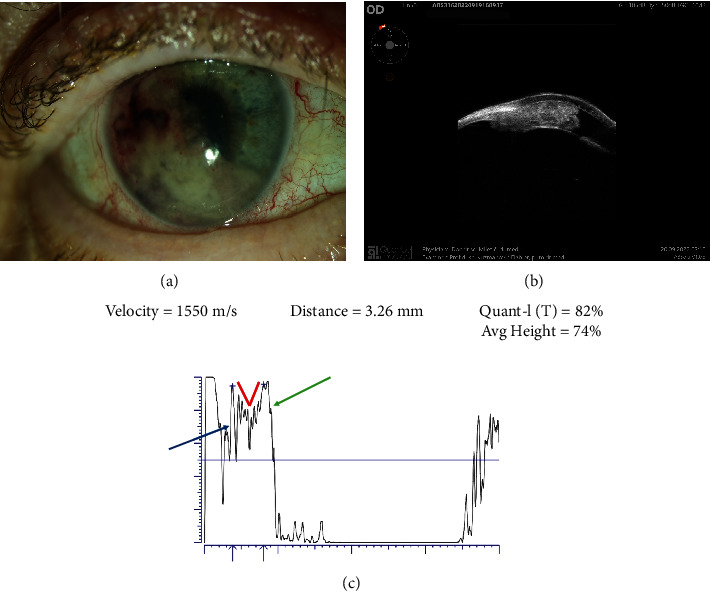
(a) Slit-lamp photography of the RE anterior segment. (b) The L-9 UBM scan (RE). (c) The immersion technique standardized A-scan echography; red lines mark the “high V“pattern; the corneal spike in front of the tumor surface pointed at with the blue arrow; the lens capsule spike behind the tumor surface marked with the green arrow.

In September 2022, an 82-year-old male presented at the Neurology Emergency Department with deterioration of vision, intense pain, and grittiness in the right eye accompanied by periocular headache since the day before. Convulsive elements, nausea and vomiting, dizziness, trauma, and photophobia were not present. The patient's past medical history was notable for myocardial infarction 10 years ago, hypertension, and hyperlipidemia. He has never smoked cigarettes. The neurological examination indicated no apparent issues. However, upon assessment, swelling was observed in the right eyelid, and the right eye appeared visibly inflamed, with a discernible mass present in the anterior chamber, even noticeable without magnification. The headache was assumed to be due to secondary glaucoma, and he was referred for an ophthalmological examination. With his right eye, he only discerned hand movements and the visual acuity of his left eye was 0.2 logMar. Intraocular pressure was 35 mmHg in the right eye and 19 mmHg in the left eye. Slit–lamp biomicroscopy revealed a ciliary injection and hemorrhagic mass overlying the temporal half of the iris of the right eye. One-third of the pupil was visible (Figure 1(a)).

A fundus examination of the right eye could not be performed. The remainder of the exam, including the contralateral eye, was unremarkable. The presence of elevated intraocular pressure, mild ciliary injection, and extensive hemorrhage suggested that this condition was not usual presentation of the typical spectrum of infectious or noninfectious uveitis. This finding, along with the anamnestic data, aroused the suspicion that other clinical entities should also be considered. Ocular echography was indicated in order to visualize previously difficult-to-reach ocular structures.

**Figure 2:**
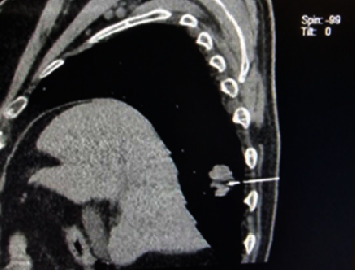
CT chest imaging.

The L-9 ultrasound biomicroscopy (UBM) scan documented the tumor completely infiltrating the iris and the ciliary body adjacent to the lens. It was easily demarcated from the overlying cornea which had increased reflectivity and irregular structure pertinent to the edema. The tumor filled the anterior chamber, the angle, and the ciliary sulcus and covered two-thirds of the pupil (Figure 1(b)). It had an irregular structure with low-reflective and highly reflective oval islands. The highest diameter measured perpendicular to the cornea and including the cornea was 3.34 mm. The immersion technique standardized A-scan echography confirmed it to be a highly reflective lesion with internal reflectivity of 82%. The tumor is the densest in the center, so the image has the lowest reflectivity in the center. Toward the normal orbit which is highly reflective, the density of the tumor decreases, and the spikes are increasing on both sides of the tumor center, creating the V-shape pattern. This pattern is, therefore, found in all sound beam directions of the tumor. The “high” part of the high V is pertinent to the high reflectivity of the tumor due to the tumor structure having the large clumps of the tumor cells. The “V” part (red lines), on the other hand, stands for the infiltrative growth of the tumor. Regarding the lens echo, the ultrasound beam has not been perpendicular either to the cornea (the low spike in front of the tumor surface pointed at with the blue arrow) or to the lens capsule (the spike behind the tumor surface pointed at with the green arrow) (Figure 1(c)). Based on previously described findings, the patient underwent a thorough investigation including laboratory testing, multislice computed tomography (MSCT) of the brain, thorax, abdomen, and pelvis. During investigations and observation at the Ophthalmology Department, intraocular pressure was maintained within target values with topical prostaglandin derivatives, beta-blockers, carbonic anhydrase inhibitors, and alpha-2 agonists. CT chest imaging showed a solid expansive formation with a longer diameter of 26 mm in the parenchyma of the posterobasal segment of the right inferior lung lobe without signs of locoregional dissemination (Figure 2). In the laboratory tests, significantly elevated values of the tumor marker CEA and Cyfra-e 21-1 were verified.

The patient was transferred to the Interventional Pulmonology Department where a fine needle aspiration biopsy (FNAB) was immediately performed under the control of MSCT (Figure 2). Cells were TTF-1 positive, CK7 positive, and CK 20 negative which confirmed primary lung adenocarcinoma. Targeted and symptomatic treatment was indicated. The samples were sent for molecular analysis and the determination of predictive biomarkers. The findings of EGFR, ALK, and ROS1 mutation later come back negative and PD-L1 expression is less than 1%. Unfortunately, 48 h after hospitalization, the patient was rehospitalized in the Internal Medicine Clinic due to SARS-CoV-2 infection with severe COVID-19 pneumonia. Despite all targeted, symptomatic and supportive treatment measures taken, the patient died in the first half of October 2022.

## 3. Discussion

During the patient's hospital stay at the Ophthalmology Department, the UBM and standardized A-scan echography proved to be invaluable in detecting any signs of metastatic disease. Standardized A-scan echography confirms structure irregularity in height, length, and width of tumor spikes. The V-shaped pattern or the high V was originally described by Prof. Ossoinig for a metastatic carcinoma of the anterior orbit (videos given at the Standardized Echography Course organized by Prof. Ossoinig). The high V pattern has been described for the large choroidal metastases but, to the best of our knowledge, not for ciliary body metastases [5–7]. However, regular infiltrative carcinoma, regardless of the location, may show the same echographic pattern. Such information is invaluable when choosing further diagnostic procedures and treatment modalities.

Between 66% and 97% of individuals diagnosed with intraocular metastasis are already treating the underlying disease and other distant metastatic lesions. However, one-third of patients may have the first manifestation of metastatic disease in the eyes, which is only suspected by a comprehensive eye examination. The ophthalmologist should suspect breast cancer, lung adenocarcinoma, and gastrointestinal tract carcinoma, which, according to the literature, have been proven to be the most common primary foci [4]. Quite often, patients have bilateral uveal metastases (18%) [8]. In a large retrospective study by Shah et al. [8], it was confirmed that the metastatic lesion in the anterior uvea is located in only 12% of cases, while in fact, the most common intraocular focus is the choroid (88%). Although these percentages support the establishment of screening methods for metastatic eye disease, the results so far show an extremely low incidence rate when it comes to lung adenocarcinoma [3]. Modern imaging techniques such as ultrasound diagnostics, anterior OCT, CT scan, and MRI play a fundamental role in the diagnosis of invasive metastatic lesions in the eye.

Treatment options for metastatic disease in the uvea are divided into local, such as external beam radiotherapy (EBRT), plaque radiotherapy, surgical excision or enucleation, and systemic, which includes systemic chemotherapy and monitoring the effect of chemotherapy on the affected eye. Modern chemotherapy and immunotherapy have led to a better quality of life for these patients [9, 10]. There are isolated cases that showed regression of metastatic uveal disease after the application of intravitreal anti-VEGF therapy (bevacizumab), which is currently proposed as an additional symptomatic therapy [11, 12]. The selection of the most effective treatment modality depends on the anatomical position of the ocular metastasis as well as other distant metastases, the characteristics of the primary tumor, and the anticipated life expectancy of the patient [13]. Nguyen et al. [13], based on previous research, found that metastases originating from the breast or lungs are very often radiosensitive and will respond well to EBRT. They also pointed out that bevacizumab could find its place in the treatment of ocular metastases in patients with colon adenocarcinoma, but due to the fear of the local spread of the disease during the application procedure, it was not widely used. There were also some case reports that described regression of ocular metastases in patients with ER–positive breast cancer treated with the hormone therapy (e.g., tamoxifen) [14, 15].

Specifically speaking of lung adenocarcinoma, genetic and molecular analysis is increasingly performed to determine EGFR mutation positivity in order to direct to EGFR tyrosine kinase inhibitor (TKI) treatment, whereby the third generation EGFR-TKI (osimertinib) is actively being researched in combination with the intravitreal bevacizumab [16]. The eye symptoms can regress in 50% of cases, but the 1-year mortality of these patients is unfortunately extremely high, and according to literature [17], it ranges between 54% and 87%. Despite the previously mentioned discouraging results, one case study showed that visual acuity of the affected eye and anterior segment exam was improved, intraocular pressure was reduced, and dilated retina exam was clear after using topical pressure lowering medications, steroid therapy, and pupillary dilating agents [18]. Although metastases in the eye are the initial manifestation of Stage IV malignancy and the prognosis is poor, local symptomatic therapy, in combination with other treatment modalities, is mandatory in an attempt to improve the patient's quality of life.

Considering the panel of predictive biomarkers, enucleation would have been indicated in our patient in order to control the local symptom of pronounced pain, which required the use of a high dose of opioid analgesics in addition to the fact that the focal orbital change drastically affected the quality of life. Considering the performance status (PS ECOG 2), the patient would have been a candidate for treatment with chemotherapy according to the pemetrexed-carboplatin protocol.

## 4. Conclusion

A multidisciplinary approach of several medical professions is needed in order to establish the diagnosis as early as possible and to optimize the possibilities of treatment of metastatic tumors to the uvea which is mostly aimed at the treatment of the primary disease.

## Data Availability

The case report data used to support the findings of this study are included within the article.
